# Does dental treatment bring health to high‐risk people with recurring disease?

**DOI:** 10.1002/jdd.13762

**Published:** 2024-10-29

**Authors:** David C. Johnsen, Leonardo Marchini, Karin Weber‐Gasparoni, John Warren, Carlos Garaicoa‐Pazmino, L. Brendan Young, Clark M. Stanford, Aditi Jain, Heidi Steinkamp

**Affiliations:** ^1^ Pediatric Dentistry, College of Dentistry University of Iowa Iowa City Iowa USA; ^2^ Preventive and Community Dentistry, College of Dentistry University of Iowa Iowa City Iowa USA; ^3^ Periodontics, College of Dentistry University of Iowa Iowa City Iowa USA; ^4^ Research Center School of Dentistry Universidad de Especialidades Espiritu Santo Samborondón Ecuador; ^5^ Prosthodontics, College of Dentistry University of Iowa Iowa City Iowa USA; ^6^ Operative Dentistry, College of Dentistry University of Iowa Iowa City Iowa USA

## 1 INTRODUCTION

A conundrum for the individual faculty or student and a conundrum for schools is the dual mission to bring oral health to its patients and at the same time train students with an array of preventive and reparative procedures for practice. This essay will offer the perspective that caring is just as important as curing and that without caring, curing is less likely. The purpose of this essay is to explore the roles and limitations of individual faculty, students, and dental schools to assist people/patients to bring health to patients, while training students in preventive and reparative procedures for practice.

A key question: “Does treatment bring/stabilize health?.”^1^ For healthy people—those who know about prevention, avoid sugar and tobacco, attend regular check‐ups, and enjoy domestic stability, financial security, and transportation—the answer can be “Yes!.” Occasional disease recurrences can be alleviated with treatment, and in this situation, dentists help patients to maintain their oral health. For the healthy person, the patient/person is self‐caring, and the occasional curing takes place with help from the student/dentist.

For groups of higher risk people with recurring disease, we do not have proven interventions to bring sustained oral health.^2^, ^3^ In short, as dentists, we have a much harder time helping patients with significant oral health problems to achieve health. Preventive and reparative procedures for higher risk individuals with recurring disease tend to mitigate rather than eliminate or even control disease.^4^ For the higher risk person who is unable to provide enough self‐caring with circumstances largely beyond their control, or who is unwilling to provide enough self‐caring, the challenge for the student/dentist to provide curing increases greatly. The next parts of the essay explore dilemmas for the individual faculty and students in trying to bring sustained health to their patients followed by dilemmas for the institution attempting to bring health to the people being served, while at the same time, training students in preventive and reparative procedures.

## 2 MAIN SECTION

Dilemmas for the individual faculty and student can start with the question from the student: “How can I bring sustained health to my patient who has recurring disease?” The student question on achieving health is a different kind of question from: “How can I make an ideal crown for this patient?” One focuses on the procedure, and one focuses on the person. We are unaware of a standard set of options for the faculty interacting with this student for higher risk people with recurring disease. All dental schools have patients who return year after year with progressing disease requiring ever escalating/switching interventions. A basic tenet of dentistry is treating the disease that presents with the patient, so that is a given, but what are the long‐term options for helping the patient with significant oral disease to achieve oral health? The recurring theme is curing starts with caring.

Considerations for faculty/students would include: (1) assessing the upstream determinants of the individual and their life situation; (2) analysis of low income as a risk factor in the disease, and how low income may affect food choices, availability of preventive regimens and the priority level of oral health among other needs; (3) the mindset of treating caries and periodontitis as chronic diseases, and appropriately managing the disease rather than only treating acute episodes; and (4) related to #3, avoiding escalating interventions for higher risk people with recurring disease.

One point central to this discussion is to address the *dentist's focus in addressing disease*. Is the focus only on mitigating the consequences of the disease—for example, with restorations or periodontal therapy (scaling and root planing [SRP]/periodontal surgery)—where success and failure are measured on the technical accomplishment. Or is the attention directed toward not only on acute management of the disease, but also on strategies that will prevent future recurrence and allow the patient to achieve and maintain good oral health? That is, are we truly focused on managing the person? A key moment for the student/dentist is assessing the patient's/person/s capacity for self‐caring. Metrics are limited for assuring the patient's capacity to subscribe to professional recommendations with a timeline for disease control/mitigation. Nor is there a set of metrics for the student/practitioner to follow/assess how well the patient adheres to professional recommendations and establishes health, and to what degree the patient cannot adhere to professional recommendations due to upstream factors that are traditionally not addressed in dental practices or educational institutions.

*Upstream determinants* for higher risk people with recurring caries or periodontitis include such things as finances, transportation, a difficult family dynamic, housing instability, lack of a support system, behavioral limitations, disability, medical conditions, and food insecurity.^5^ In brief, the critical factor is the patient's *capacity to subscribe to professional recommendations*.^6^ Capacity in this context refers to the physical, emotional, and socioeconomic capability and the *means* necessary to change course and adhere to a healthier lifestyle. If the patient has the capacity to subscribe to professional recommendations, sustained health is attainable. If not, the risk escalates. The clinician's assessment of that capacity for the patient's/person self‐caring is central to determining the patient's future health (prognosis).

**FIGURE 1:**
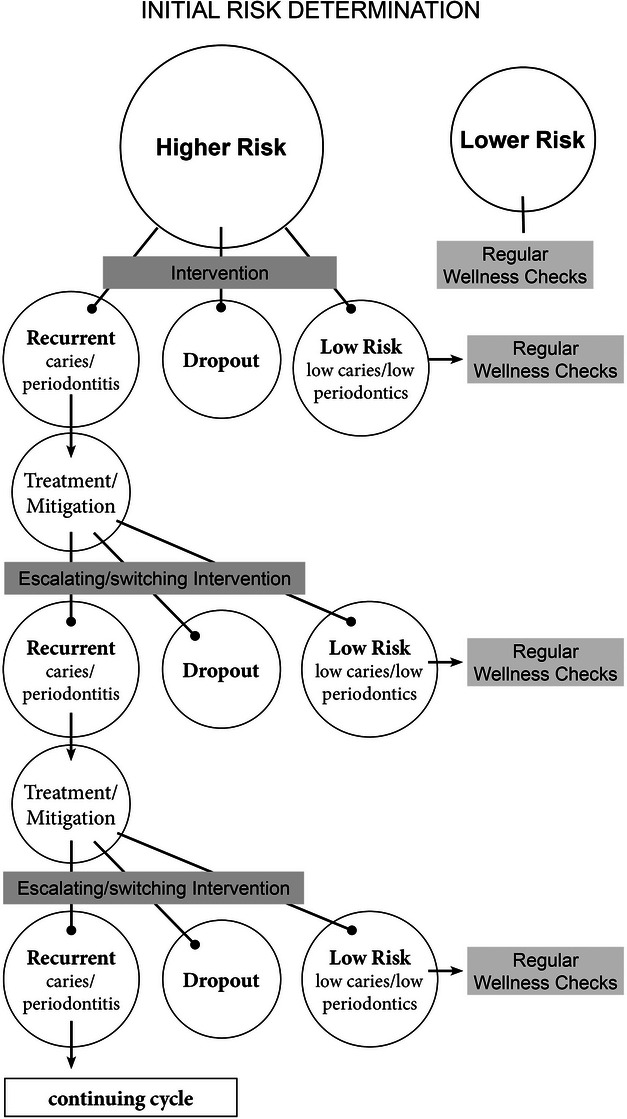
Schematic showing escalating or switching interventions for higher risk people with recurring disease.

Without addressing these upstream determinants, there is great risk of *escalating or switching interventions* in patients with recurring disease (Figure 1). For example, at the outset, the practitioner treats the existing condition, and makes professional recommendations to bring health. Some patients will comply, and some will not or do not have the capacity to do so. On the next round, with continued or recurring disease, the professional recommendations intensify or switch course, again with some patients adhering and some not. The student's/dentist's ability to reassess the person's/patient's capacity for self‐caring is accentuated at each escalating intervention. For the nonadherent people, the interventions intensify again, and the cycle goes on. Such escalating treatment can be expensive for the patient, frustrating for the dentist, and ultimately does not bring health to the patient. Moreover, fatigue and discontinuation of care can follow for the patient and the dentist.

A *supplement* is added to illustrate a dynamic for escalating/switching interventions for high‐risk people. The scenario is a synthesis of some years of patient experiences and does not represent one or a small number of patients.

Caries and periodontitis are chronic conditions. If we frame caries and periodontitis only as acute, then treatment requires little involvement of the patient—patients simply go to the dentist to get something “fixed.” If we frame caries and periodontitis as chronic, treatment is unlikely to bring health without full engagement/self‐caring of the patient.^7^ Much of dentistry involves treatment with limited sustained engagement of the person/patient. For the healthy person who already knows a good deal about maintaining good health, viewing caries, and periodontitis as acute diseases with occasional treatment can continue to bring sustained health. For the compromised or noncompliant person, it will be more important to view caries and periodontitis as chronic diseases with active engagement between the dental team and the person/patient, including acceptance of limitations to a highly structured regimen. While many experienced practitioners have adapted to addressing upstream factors related to self‐caring to some degree, the challenge for dental education is what additional training may be necessary to more effectively managing a person with a chronic disease to restore health. For example, how early should students be introduced to these concepts and what disciplines should be included? Should courses on managing chronic disease or mitigating social factors be required? The theme of curing starting with caring can be part of a curriculum (Supporting Information S1).

*It is well known that lower resourced individuals* have higher rates of caries and periodontitis as a group, but clearly the lower income alone is not the direct cause of these diseases.^8^ Thus, the focus must be on reducing the barrier of lower income, in order for low‐income people to become healthy. This will require addressing other individual upstream determinants that contribute to higher disease and compromised health. For example, dentists can promote healthy foods (and reducing sugar consumption) by providing information on government programs (e.g., Supplemental Nutrition Assistance Program [SNAP] for Women, Infants and Children [WIC]), and food banks to patients. Taken further, dentists and dental schools should advocate for expansion of these programs and things such as free school lunches. Dental schools can also work with, support and advocate for programs that support families help them to manage stressors. Other factors to address include medical conditions, disability, entrenched behaviors, lack of a social support net, remoteness from care, and lack of health insurance. The point of this is that dental students need to be made aware of upstream factors and appropriate interventions that have a chance to help lower income people achieve health.^9^

It should be stressed that procedures to restore teeth are one strategy that seems unlikely—by itself—to allow low income or disabled people to achieve health. While training dental students to restore teeth is essential, it is not sufficient training to help those with chronic oral disease to achieve health. Curing starts with caring from the student/dentist and patient/person. Another important component to helping people achieve health is effective communication. Communication strategies include a qualitative component and a quantitative component. The qualitative component involves the methods for communication. The quantitative component involves how extensively we need to engage the patient to bring behavior change—and the literature on the quantitative component is sparse. *Communicative strategies* (the qualitative component) can include:

1. Reinforcing effective behaviors with praise (e.g., coming in for an exam).
2. Describing both the positive and problematic oral conditions.
3. Explaining the causes of the problematic conditions.
4. Describing the consequences of doing nothing and acknowledging the possibility of failure.^10^

It is also important to adapt messaging to the patient, using noncontrolling, nonjudgmental language with empathy and respect, fully respecting patient autonomy, and allowing their voices to be heard.

For the quantitative component of an intervention, one line of discussion can be: “We have interventions that can bring health to your patient. We must try! We do not know the level of engagement needed to find the person's level of self‐caring and bring behavior change to then bring health for your patient. We will have failures. We must try again!.”

*Dental institutions have a moral obligation* in training and educating students to maintain, stabilize, and ultimately restore health. Academic health centers are uniquely positioned to address health, and dental schools can lead the effort for dentistry. So how does the mission of bringing health line up with what/how dental education is delivered for our students? Dental education has a large component focused on demonstration of competency in delivering procedures aimed at treating/mitigating oral diseases with an appropriate focus on the two most common oral diseases of caries and periodontitis. Accreditation has a detailed list of expected procedures.

## 3 SUMMARY

Two different kinds of questions are appropriately asked by our students. The first is from the student with a patient who is at higher risk with recurring disease, who asks the faculty member: “How can I assist my patient to achieve sustained health?” The focus is on the person. For the second kind of question from the student: “How can I make an ideal crown for this patient?,” the focus is more on the tooth/teeth. Both are essential and follow fundamentally different lines of thinking. A central theme of this essay is that caring is essential for curing. For the healthy person, with a high capability for self‐caring and knowledgeable about prevention the focus on the tooth/teeth may be enough. For the higher risk person for whom self‐caring may be beyond their capacity for self‐caring through no fault of their own or who are unwilling to provide self‐caring, the challenge to the student/dentist increases greatly, and a focus on the teeth alone will lead to recurring disease. Thus, dental schools can move toward strategies to address and mitigate upstream factors for many patients to *achieve* health.

## 4 SUPPLEMENTAL MATERIAL

The following is an example of escalating/switching interventions for a higher risk person. The scenario is a synthesis of some years of patient experiences and does not represent one or a small number of patients:

A young adult patient with multiple mental health diagnoses (post‐traumatic stress disorder [PTSD], general anxiety, and depression) and corresponding medications came for an initial consultation. The patient presented with multiple carious lesions and significant plaque accumulation. After the initial examination, it was determined that the patient had a high caries risk from frequent sweet soda drinks, but the teeth were restorable. The patient received standard oral hygiene instructions (OHI), a prescription for 1.1% neutral sodium fluoride brush‐on gel (Colgate® PreviDent®), a prophylaxis, counseling on sweet restriction, and a plan for restorative treatment, followed by a 6‐month recall schedule.

After a couple of years without returning, the patient came back for an emergency appointment with an abscessed tooth, multiple carious lesions, and significant plaque accumulation. At this point, a few teeth were not restorable, and the patient required extractions, prophy, operative care, and prosthodontic treatment (maxillary and mandibular removable partial denture [RPD]). This time, however, the provider took the time to listen to why the patient was not following OHI. Instead of lecturing the patient, the provider listened to the patient's reasons and offered a few options to help address those concerns. The provider asked the patient to start by sweet restriction, brushing once a day, initially at night, and using Prevident®. The patient agreed.

At the next visit, some improvement was noted. The patient was praised, and some fillings were completed. The patient complained of dry mouth for the first time. The provider expressed sympathy and offered several options to reduce dry mouth sensation, switched to a dry mouth formulation of Prevident®, and completed some operative work, including in an esthetic area. The patient was pleased with the attention and the improvement in their smile. The appointments continued with increasing trust and consequently, adherence to recommendations. The maxillary and mandibular RPDs were delivered, and the patient began recalls. After missing the first recall, the provider called and found that the patient was experiencing a depressive episode. The provider contacted the primary care provider, adjustments were made to the patient's antidepressants, and the patient's condition improved. The provider called again and rescheduled the recall. After noting significant plaque accumulation, sweet restriction counseling, and a prophylaxis were done. The provider asked how the patient was feeling and suggested restarting with brushing before sleep using Prevident®. The patient agreed, and they decided on a 3‐month recall schedule until the depressive episodes stabilized. And so, it goes.

## Supporting information



Supporting Information
