# Supplementary material for: Does dental treatment bring health to high‐risk people with recurring disease?
Source: J Dent Educ. 2024 Oct 29;89(4):537–42. doi: 10.1002/jdd.13762 (PMC12004341; doi:10.1002/jdd.13762)
Supplement: Supplementary file 1 — Supporting Information [file JDD-89-537-s001.docx]

JDETxHealthEdSupplmt24

**Supplemental material**

The following is an example of escalating/switching interventions for a higher risk person. The scenario is a synthesis of some years of patient experiences and does not represent one or a small number of patients:

A young adult patient with multiple mental health diagnoses (post-traumatic stress disorder [PTSD], general anxiety, and depression) and corresponding medications came for an initial consultation. The patient presented with multiple carious lesions and significant plaque accumulation. After the initial examination, it was determined that the patient had a high caries risk from frequent sweet soda drinks, but the teeth were restorable. The patient received standard oral hygiene instructions (OHI), a prescription for 1.1% neutral sodium fluoride brush-on gel (Colgate® PreviDent®), a prophylaxis, counseling on sweet restriction, and a plan for restorative treatment, followed by a 6-month recall schedule.

After a couple of years without returning, the patient came back for an emergency appointment with an abscessed tooth, multiple carious lesions, and significant plaque accumulation. At this point, a few teeth were not restorable, and the patient required extractions, prophy, operative care, and prosthodontic treatment (maxillary and mandibular removable partial denture [RPD]). This time, however, the provider took the time to listen to why the patient was not following oral hygiene instructions. Instead of lecturing the patient, the provider listened to the patient's reasons and offered a few options to help address those concerns. The provider asked the patient to start by sweet restriction, brushing once a day, initially at night, and using Prevident®. The patient agreed.

At the next visit, some improvement was noted. The patient was praised, and some fillings were completed. The patient complained of dry mouth for the first time. The provider expressed sympathy and offered several options to reduce dry mouth sensation, switched to a dry mouth formulation of Prevident®, and completed some operative work, including in an esthetic area. The patient was pleased with the attention and the improvement in their smile. The appointments continued with increasing trust and consequently, adherence to recommendations. The maxillary and mandibular RPDs were delivered, and the patient began recalls. After missing the first recall, the provider called and found that the patient was experiencing a depressive episode. The provider contacted the primary care provider, adjustments were made to the patient's antidepressants, and the patient's condition improved. The provider called again and rescheduled the recall. After noting significant plaque accumulation, sweet restriction counseling and a prophylaxis were done. The provider asked how the patient was feeling and suggested restarting with brushing before sleep using Prevident®. The patient agreed, and they decided on a 3-month recall schedule until the depressive episodes stabilized. And so, it goes.
